# Systemic therapies in hepatocellular carcinoma: Existing and emerging biomarkers for treatment response

**DOI:** 10.3389/fonc.2022.1015527

**Published:** 2022-11-22

**Authors:** Penghui He, Haifeng Wan, Juan Wan, Hanyu Jiang, Yu Yang, Kunlin Xie, Hong Wu

**Affiliations:** ^1^ Department of Liver Transplant Center, West China Hospital, Sichuan University, Chengdu, Sichuan, China; ^2^ Department of Pancreatitis Center, West China Hospital, Sichuan University, Chengdu, Sichuan, China; ^3^ Department of Radiology, West China Hospital, Sichuan University, Chengdu, Sichuan, China; ^4^ Department of Abdominal Oncology, Cancer Center, West China Hospital of Sichuan University, Chengdu, China

**Keywords:** hepatocellular carcinoma, predictive biomarker, systemic treatment, molecular targeted therapy, immunotherapy

## Abstract

Hepatocellular carcinoma (HCC) is the fifth most common malignancy and the third most common cause of cancer-related death worldwide. Due to asymptomatic patients in the early stage, most patients are diagnosed at an advanced stage and lose the opportunity for radical resection. In addition, for patients who underwent procedures with curative intent for early-stage HCC, up to 70% of patients may have disease recurrence within 5 years. With the advent of an increasing number of systemic therapy medications, we now have more options for the treatment of HCC. However, data from clinical studies show that with different combinations of regimens, the objective response rate is approximately 40%, and most patients will not respond to treatment. In this setting, biomarkers for predicting treatment response are of great significance for precise treatment, reducing drug side effects and saving medical resources. In this review, we summarized the existing and emerging biomarkers in the literature, with special emphasis on the pathways and mechanism underlying the prediction value of those biomarkers for systemic treatment response.

## 1 Introduction

Liver cancer is the third most common death-related malignant tumor in the world, and China bears the brunt of it with the highest number of deaths annually (1). Hepatocellular carcinoma (HCC) represents the most frequent histologic type of primary liver cancer, accounting for 75%-85% of cases (1). Due to the early asymptomatic period, most patients are diagnosed at an advanced stage. Even for early-stage HCC patients, the recurrence rate after surgical resection remains high (2, 3).

In recent years, a growing number of studies have focused on the systemic treatment of HCC. Targeted therapy and immunotherapy have played an important role in the combined treatment of HCC. Tyrosine kinase inhibitors (TKIs) targeting pathways involved in the amplification and proliferation of tumors (4). Since sorafenib was approved as the first molecular targeted agents (MTAs) for the treatment of advanced HCC in 2007 (5), molecular targeted therapy has made rapid progress. With a growing number of clinical studies being carried out, sorafenib and lenvatinib have been approved as first-line MTAs, and regorafenib, cabozantinib, anlotinib and ramucirumab have been approved as second-line MTAs. The general mechanism of immunotherapy is to enhance anticancer immunity in the tumor microenvironment by blocking the negative feedback pathway of the immune system (6). Programmed death-1 (PD-1)/programmed death-ligand 1 (PD-L1) and cytotoxic T lymphocyte-associated antigen-4 (CTLA-4) are major molecules involved in suppressing the immune response (6, 7). Although studies have demonstrated the effectiveness of systemic therapy in improving the prognosis of HCC patients (8), its limited response rates are still a bothersome issue to be solved. By monotherapy, the response rate to sorafenib is less than 5% (9), and the response rate to immunotherapy is less than 20% (10, 11). A low response rate prevents the majority of HCC patients from receiving treatment benefits. Therefore, biomarkers that can aid in the selection of HCC patients who respond to systemic therapy are critical. In this review, we summarized potential biomarkers of response to systemic therapy in HCC.

## 2 Clinical factors

### 2.1 Aetiological factors

As a multicausal disease, the causes of HCC include hepatitis B virus (HBV) infection, hepatitis C virus (HCV) infection, chronic alcohol intake, and nonalcoholic steatohepatitis. Different etiologies represent different tumorigenesis mechanisms and may have different susceptibilities to antiangiogenic drugs. A recent retrospective study performed by Tomonari et al. showed that in HCC patients treated with lenvatinib, the objective response rate (ORR) was higher in the nonviral group than in the viral group, although the difference was not significant. While the progression-free survival (PFS) and overall survival (OS) was significantly longer in the nonviral group than the viral group, suggesting that nonviral status might serve as a biomarker for lenvatinib treatment (12). The authors held that fibroblast growth factor 19 - fibroblast growth factor receptor 4 (FGF19-FGFR4) pathway, which was target of lenvatinib, were involved in the tumorigenesis of non-alcoholic steatohepatitis- and alcohol-associated HCC and consequently, these HCCs responded better to lenvatinib. Moreover, in a meta-analysis, Shao et al. reported that among sorafenib-treated HCC patients, both HCV-positive and HCV-negative patients had a significantly lower risk of death than controls (13). In addition, the benefit from sorafenib in HCV-positive patients was significantly greater than that in HCV-negative patients (Hazard ratios were 0.65 versus 0.87). And Kolamunnage-Dona et al. suggested that sorafenib was associated with reducing tumor growth rate and deterioration of liver function of HCV-induced HCC patients (14). The underlying mechanism might be as follow. The upregulation of Raf and downstream signaling could be induced by the transcription regulation of HCV core (15). And Raf signaling pathway represents one of sorafenib targets. Moreover, miRNA-dependent modulation of Mcl-1 by HCV protein enhances sorafenib sensitivity (16). These results suggested that HCV-positive patients may have a better response to sorafenib treatment. These studies indicated that nonviral-associated HCC or HCV-associated HCC responded better to first-line TKIs. However, HBV infection represents the major etiology of HCC patients, and how to improve the response rate of HBV-associated HCC patients to sorafenib and lenvatinib treatment still needs further investigation.

### 2.2 Lung metastasis

The lung is the predominant location for extrahepatic metastasis of HCC, accounting for more than 40% of HCC (17–19). Studies have been conducted to investigate the response to systemic therapy of HCC with lung metastasis. Yau et al. performed a phase II open-label trial of sorafenib monotherapy treating advanced HCC in an Asian population with prevalent hepatitis B. They found that patients with lung metastases had a worse clinical benefit than those without lung metastases. The authors speculated that this could be due to the distant metastasis of HCC itself being a poor prognostic factor (20). In another retrospective study, sorafenib responders had a higher rate of lung metastases than those who did not respond to sorafenib, although the difference was not statistically significant. However, considering the number of lung metastases, the incidence of multiple lung metastases (n ≥ 5) in responders was significantly higher than that in non-responders (21). An in-depth study of HCC molecular subtyping has provided clues to this phenomenon, reporting that macrotrabecular-massive HCC, characterized by aggressiveness with both angiopoietin 2 (Ang-2) and vascular endothelial growth factor A (VEGFA) overexpression, with a hallmark feature of angiogenesis, was the most common subtype with a high potential for metastasis (22) (23). And the angiogenesis could be promoted by VEGFA through Raf/mitogen-activated protein kinase pathway, which is one of the major target of sorafenib.

On the other hand, studies on HCC immunotherapy have reported consistently favorable results for lung metastasis compared with the primary tumor. Lu et al. reported that intrahepatic lesions of HCC were less responsive to immune checkpoint inhibitors (ICIs) than extrahepatic lesions, with lung metastases most positively responding to ICIs (24). The distinct tumor microenvironment between the liver and the lung may underlie the difference in treatment response. The lung tumor microenvironment is reported to be richer in immune cells than other organs (25, 26). In contrast, immunosuppressive cells in the liver can contribute to the immunosuppressive microenvironment of the organ (27). Moreover, tumor volume is another factor affecting the response to immunotherapy. Huang et al. reported that, small HCCs had more immune cell infiltration than large HCCs, including CD8+ T cells, M1 macrophages, and monocytes, and small lesions had better sensitivity to ICIs than large lesions (28). As lung metastasis, in most cases, is relatively smaller than primary liver tumors, this may explain the difference in ICI therapy response between the primary and lung metastases of HCC.

### 2.3 Adverse events

For molecular targeting therapy, AEs mainly manifest as skin toxicity, digestive system reactions, and hypertension. Vincenzi et al. retrospectively analyzed the association between skin toxicity (rash and hand-foot skin reaction (HFSR)), disease control rate (DCR), and time to progression (TTP) in HCC patients receiving sorafenib and found that for patients with skin toxicity during treatment, both DCR and TTP were better than those without (29). A meta-analysis by Wang et al. found that patients who developed HFSR during sorafenib treatment had better TTP than those who did not (30). In addition, studies further investigated the predictive value of different HFSR grades for the efficacy of sorafenib and found that HFSR grade ≥ 2 was the most favorable predictor of response (31–33).

The digestive system AEs mainly manifested as diarrhea. In a retrospective study, Cho et al. found that the presence of HFSR and diarrhea were correlated with a prolonged TTP for HCC patients receiving sorafenib (34). Likewise, combining patients with partial response (PR) and stable disease (SD), HFSR and diarrhea were found to be predictors of sorafenib response (35).

Hypertension is also a common side effect of molecular targeted therapy. Considering the similarity between the mechanism of hypertension and the antitumor mechanism of sorafenib, Van Leeuwen et al. believed that the development of hypertension is a pharmacodynamic marker of treatment efficacy (36). Yang et al. reported the superiority of TTP in HCC patients with hypertension over those without hypertension during apatinib treatment (37). In addition, Lee et al. suggested that HFSR, diarrhea, and hypertension have predictive value in response to sorafenib, and with the increase in the number of AEs, TTP and OS were improved (38).

Immune-related adverse events (irAEs) have also been proposed as a predictive biomarker for immunotherapy response. Lu et al. showed that HCC patients with irAEs (mainly rash) had a significantly higher tumor response rate and DCR than those without irAEs when treated with anti-PD-1 antibodies (39).

The TKI-associated AEs were reported to be probably induced by the inhibition of vascular endothelial growth factor receptor and platelet-derived growth factor receptor (40, 41). As for irAEs, the underlying mechanism is still being explored. The cross-reactivity of immune response between tumors and normal tissue might provide us some clues (42). Besides, Hinrichs et al. proposed that healthy tissue might have molecular mimicry expressed, which are antigens identical to tumor antigens. When treated with ICIs, those healthy tissue may have immune response similar to that of tumor (43). Therefore, both TKI-associated AEs and irAEs could be considered as external manifestation of agents effect. Given that these AEs are potentially predictive biomarkers for systemic therapy response, it is important to encourage patients to adhere to their medication while managing AEs. **Table 1** summarized the clinical factors predictive of response to systemic treatment for HCC.

**Table 1:** Clinical Predictive factors for the systemic treatment of hepatocellular carcinoma.

Clinical factors	Reference	Hazard ratio (95% confidence interval)		Positive or Negative
		PFS, TTP or PD	OS	
Nonviral status	Tomonari et al. (12)	0.324 (0.174–0.602)	0.277 (0.116-0.662)	Positive for lenvatinib
HCV	Shao et al. (13)	0.65 (0.53–0.80) versus 0.87 (0.79–0.96) of HCV+ versus HCV- for OS		Positive for sorafenib
Kolamunnage-Dona et al. (14)		NA	NA	
Lung metastasis	Arao et al. (21)	NA	NA	Positive for sorafenib
	Lu et al. (24)	NA	NA	Positive for ICIs
(immune related) Adverse events				
Skin toxicity	Vincenzi et al. (29)	0.412 (0.176 – 0.820)	NA	Positive for sorafenib
HFSR	Wang et al. (30)	0.41 (0.28-0.6)	0.45 (0.36-0.55)	Positive for sorafenib
Skin toxicity	Shomura et al. (31)	NA	0.267 (0.102-0.701)	Positive for sorafenib
HSFR	Wang et al. (32)	0.74 (0.58-0.96)	0.53 (0.443-0.67)	Positive for sorafenib
	Cho et al. (34)	HSFR:0.4 (0.19-0.82)	0.4 (0.24-0.67)	Positive for sorafenib
		Diarrhea:0.34(0.15-0.74)	0.52(0.31-0.88)
Hypertension	Yang et al. (37)	0.563 (0.413–0.768)	0.520 (0.349–0.775)	Positive for apatinib
	Lu et al. (39)	0.22 (0.09-0.57)	NA	Positive for PD-1 blockade

PFS, Progression-free survival; TTP, Time to progression; PD, Progressive disease; OS, Overall survival; HCV, Hepatitis C virus; ICIs, Immune checkpoint inhibitors; PD-1, Programmed death-1; NA, Not available.

## 3 Blood biomarkers

### 3.1 Tumor markers

#### 3.1.1 Alpha-fetoprotein and Des-γ-carboxyprothrombin

Approximately 70% of HCC patients have elevated baseline levels of AFP. At present, it is mainly used as a diagnostic serum marker of primary liver cancer in clinical practice. Shao et al. studied the predictive function of early AFP response (defined as a greater than 20% decrease in AFP from baseline within four weeks of treatment initiation) for the treatment of antiangiogenic agents (44). The results showed that the early AFP responder group had a better ORR and DCR than non-AFP responders. Similar results have also been yielded by other studies (45–47). These studies revealed that early AFP changes during sorafenib treatment have the potential to help select HCC patients who might benefit from sorafenib. And AFP response phenomenon could be due to the killing of HCC tumor cells during treatment. However, AFP responses are only detected during treatment; for those without AFP responses, this may lead to a waste of medical resources and missed opportunities for treatment.

DCP is also a tumor marker for HCC diagnosis and prognosis (48–50). In a retrospective study, Ueshima et al. investigated the predictive function of DCP and found that after 2 weeks of sorafenib treatment, patients with DCP levels ≥2-fold higher than before treatment had significantly longer TTP than those without DCP elevation (51). This finding was explained by the fact that hypoxia induced by sorafenib contributes to the production of DCP; in this setting, an early increase in DCP could reflect the efficacy of sorafenib (51).

The predictive role of AFP and DCP has also been investigated in immunotherapies (52–56). As with TKI treatment, early AFP reduction after treatment initiation was also found to be correlated with objective response to immunotherapy. In contrast, reductions in DCP were associated with an objective response to immunotherapy treatment (55), indicating that posttreatment changes in DCP may have opposite predictive values when treated with TKIs or immunotherapy. This may limit its predictive power in the situation of combining TKI treatment and immunotherapy. With the predictive role of AFP and DCP being elucidated, these two tumor markers may not only contribute to the diagnosis of HCC but also help to select appropriate remedies for HCC patients.

#### 3.2 Interleukin-6/interleukin-8

**Figure 1:**
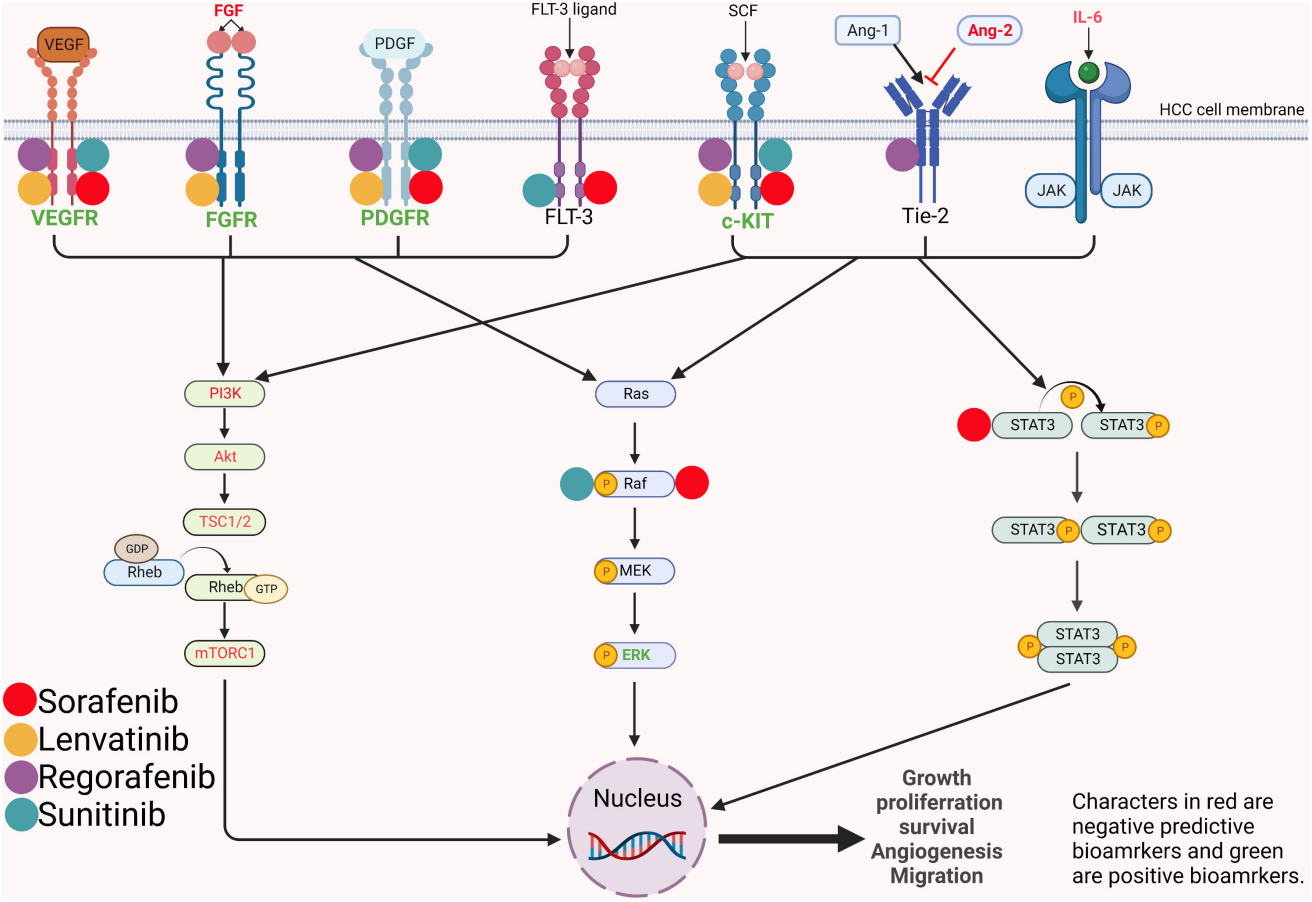
Mechanism and pathways involved in the angiogenesis and proliferation of hepatocellular carcinoma and potential predictive biomarkers. VEGFR, vascular endothelial growth factor receptor; FGFR, fibroblast growth factor; PDFGR, platelet-derived growth factor receptor; FLT-3, FMS-like tyrosine kinase 3; SCF, stem cell factor; Ang, angiopoietin; Tie2, tyrosine-protein kinase receptor; IL-6, interleukin-6; JAK, janus kinase; PI3K, phosphatidylinositol 3-kinase; Akt, protein kinase B; TSC, tuberous sclerosis complex subunit; Rheb, ras homolog enriched in brain; mTORC1, mammalian target of rapamycin complex 1; MEK, mitogen-activated protein kinase kinase; ERK, extracellular signal–regulated kinase; STAT, signal transducer and activator of transcription.

IL-6 and IL-8 are key inflammatory response mediators that promote angiogenesis. Preclinical studies have confirmed their promotion of sorafenib resistance through different mechanisms (57–59). The IL-6/signal transducer and activator of transcription 3 (STAT3) signaling pathway is involved in the angiogenesis and proliferation of HCC and STAT3 is one of the targets of sorafenib (**Figure 1**). Besides, as a cytokine derived from macrophage, IL-8 induces tumor angiogenesis and recruits immunosuppressive cells to tumors. In a nonrandomized phase II study, Boige et al. found that in patients with HCC treated with bevacizumab, low IL-8 levels at any time point were associated with better DCR (60). More recently, Öcal et al. showed that lower levels of IL-6 and IL-8 were associated with objective responses to sorafenib treatment (61). Moreover, there are also studies exploring the relationship between the changes in IL-6/IL-8 and the efficacy during treatment. Shao et al. found that patients with PD after receiving sorafenib plus tegafur had significantly higher posttreatment IL-6 and IL-8 levels than control patients (62).

IL-6 exerts both positive and negative effects on tumor immunity. It induces T-cell infiltration but also suppresses T cells by recruiting myeloid-derived suppressor cells (63–65). Myojin et al. reported that high serum IL-6 and interferon alpha were significantly associated with PD after atezolizumab plus bevacizumab treatment (66). Whether IL-6 can promote disease progression after or under immunotherapy or whether the negative immune effect of IL-6 can override the positive immune effect and appear to be related to the disease progression response after immunotherapy is worthy of further investigation.

### 3.3 Markers related to angiogenesis and proliferation

Because of the antiproliferative and antiangiogenic effects of TKIs, it is reasonable to assume that angiogenesis or proliferation-related biomarkers may to some extent reflect the therapeutic response of TKIs.

### 3.3.1 Vascular endothelial growth factors

Sorafenib blocks tumor angiogenesis by targeting vascular endothelial growth factor receptor-2/-3 (VEGFR-2/-3) and platelet-derived growth factor receptor-beta (PDGFR-beta) tyrosine kinases (67) (**Figure 1**). In a retrospective study of 30 patients (68), Miyahara et al. found that baseline cytokine levels, including VEGFA, were significantly higher in PD patients than in non-PD patients when treated with sorafenib. There was a trend of worse treatment response with more elevated baseline cytokines. Zhu et al. and Faivre et al. demonstrated that high serum VEGFC was associated with longer TTP and higher DCR under sorafenib and sunitinib treatment (69) (70). Different results suggest that VEGFA and VEGFC might have opposite predictive functions for MTAs. The divergent predictive value of VEGFA and VEGFC might be explained by their different receptors. VEGFA promotes the carcinogenesis of HCC through VEGFR-1 and VEGFR-2. While the receptors of VEGFC are VEGFR-2 and VEGFR-3, which are major targets of sorafenib and sunitinib.

### 3.3.2 Angiopoietin-2 and FGF 19/FGF 23

Normally, Ang-2 binds to its receptor Tie2 and promotes the proliferation of vascular endothelial cells to form new blood vessels in the presence of VEGF (71). In disease states, elevated Ang-2 is associated with tumor cell proliferation and may lead to vascular leakage and metastasis (72) (**Figure 1**). Ang-2 has been found to be a negative predictive factor for TKI treatment. Miyahara et al (68) described that Ang-2 had a similar predictive function as VEGFA and that Ang-2 levels were significantly elevated in patients with PD prior to sorafenib treatment. Similarly, recently, Yang et al. reported that higher baseline Ang-2 levels were significantly associated with a nonobjective response in HCC patients treated with lenvatinib (73).

FGFs are also involved in the pathogenesis of HCC. Lenvatinib is a multiple kinase inhibitor targeting VEGF receptor 1–3 (VEGFR1-3), PDGFR-α, c-Kit and FGF receptor 1-4 (FGFR1-4) (**Figure 1**). In a study evaluating multiple biomarkers, Shigesawa et al. found that in lenvatinib-treated patients, FGF 19 levels in the objective response group were significantly lower than those in the nonobjective response group (74).

Based on the REFLECT study, Finn et al. observed that increases in FGF19 and FGF23 and decreases in Ang-2 were associated with tumor response in lenvatinib-treated patients but not in sorafenib-treated patients (75). Considering that FGF19 is a ligand of FGFR4, by inhibiting FGFRs, lenvatinib treatment could lead to an increase in FGF19 through inverse feedback. Moreover, lenvatinib treatment reduced Ang-2 levels through its anti-VEGF function. Therefore, elevated FGF19 and decreased Ang-2 could represent the effect of lenvatinib and may guide therapeutic decisions in the future.

### 3.4 Circulating tumor cells and circulating tumor DNA/circulating free DNA

Since HCC diagnosis is based on radiological features, HCC tumor specimens are often not available prior to treatment. In recent years, liquid biopsy has been proposed as a new tumor biopsy technique. It is able to obtain CTCs and nucleic acids released by tumor cells.

CTCs are tumor cells isolated into the blood from primary or metastatic tumors. Tumor cells expressing PD-L1 are reported to respond better to PD-1/PD-L1 inhibitors (11). Winograd et al. found that of 10 HCC patients treated with PD-1 inhibitors, 6 had baseline PD-L1+ CTCs, and 5 of these 6 patients responded to treatment (76). The remaining 4 patients without baseline PD-L1+ CTCs showed no response.

By using liquid biopsy, Nakatsuka et al. found that HCC patients who both responded and non-responded to MTAs had an increase in cfDNA after treatment, but those who respond had a significantly greater increase in cfDNA (77). This is mainly because tumor cells killed by the treatment release tumor DNA into the plasma, resulting in an increase in cfDNA. Next-generation sequencing also confirmed tumor gene mutations in cfDNA after treatment, supporting the claim that necrotic tumor cells release DNA into plasma.

Hsu et al. investigated whether ctDNA could monitor the efficacy of atezolizumab combined with bevacizumab in HCC patients (78). They found that patients whose ctDNA level became negative during treatment had a higher response rate than patients whose ctDNA was positive.

Clinically, evaluating tumor response mainly depends on Response Evaluation Criteria in Solid Tumors (RECIST) or modified RECIST (mRECIST) criteria by radiology, but tumor size may not present evident change during treatment. Thus, the changes in ctDNA and cfDNA may better assist in screening patients suitable for systemic treatment. The aforementioned circulating predictive biomarkers were summarized in **Table 2**.

**Table 2:** Circulating predictive biomarkers for the systemic treatment of hepatocellular carcinoma.

Circulating biomarkers	Reference	Hazard ratio (95% confidence interval)		Cut-off	Positive or Negative
		PFS, TTP or PD	OS		
AFP response	Shao et al. (44)	0.307 (0.140-0.671)	0.356 (0.152-0.833)	AFP decrease by 20% within 4 weeks of treatment	Positive for antiangiogenic therapy
	Yau et al. (45)	0.31 (0.13-0.76)	0.3 (0.09-1.02)	AFP decrease by 20% after 6 weeks of treatment	Positive for sorafenib
	Shao et al. (52)	0.128(0.041‐0.399)	0.089 (0.018‐0.441)	AFP decrease by 20% within 4 weeks of treatment	Positive for ICIs
	Lee et al. (53)	NA	0.234 (0.096–0.569)	AFP decrease by 20% within 4 weeks of treatment	Positive for ICIs
	Sun et al. (55)	0.38 (0.23-0.61)	0.50 (0.32–0.80)	AFP reduction > 50%	Positive for PD-1 blockade
DCP	Ueshima et al. (51)	NA	NA	≥2-fold higher than pretreatment levels at 2 weeks	Negative for sorafenib
	Sun et al. (55)	0.60 (0.39–0.93)	0.54 (0.35–0.84)	AFP reduction > 50%	Positive for PD-1 blockade
Interleukin-6	Ocal (61)	NA	2.99 (1.22–7.3)	8.58 pg/mL	Negative for sorafenib
	Myojin (66)	2.785(1.216–6.380)	NA	4.77pg/L	Negative for Atezolizumab plus Bevacizumab
Interleukin-8	Boige et al. (61)	NA	2.19 (1.02–4.7)		Negative for sorafenib
VEGF-A	Miyahara et al. (68)	NA	NA	Mean value	Negative for sorafenib
	Zhu et al. (69)	NA	1.386 (1.119-1.715)	1162.4pg/L	Negative for sorafenib
VEGF-C	Zhu et al. (69)	0.633 (0.505-0.793)	0.829 (0.674-1.020)	906.9pg/L	Positive for sorafenib
Ang-2	Miyahara et al. (68)	2.51 (1.01–6.57)	NA	Mean value	Negative for sorafenib

PFS: Progression-free survival; TTP, Time to progression; PD, Progressive disease; OS, Overall survival; AFP, alpha-fetoprotein; DCP, Des-γ-carboxyprothrombin; VEGF, vascular endothelial growth factor; ICIs, Immune checkpoint inhibitors; PD-1, Programmed death-1; Ang-2, angiopoietin-2; NA, Not available.

## 4 Imaging features of response prediction

As the most important diagnostic tool for HCC, imaging techniques were also studied to potentially have predictive power for treatment response.

### 4.1 Imaging features of TKIs response prediction

#### 4.1.1 Dynamic contrast-enhanced ultrasound

DCE-US, by using intravenous injection of contrast agent Sonovue microbubble and vascular imaging software, is applied to accurately detect microvessles and tissue perfusion in tumors. Due to the antiangiogenic effects of TKIs, tumor vascularity is altered during MTA treatment. Therefore, DCE-US is an ideal tool to detect these changes.

In a prospective single center study, Frampas et al. found that the area under the time-intensity curve of contrast-enhanced ultrasound was significantly associated with targeted treatment response, a decrease in the area under the time-intensity curve of more than 40% at month 1 correlating to non-progression at month 2 treatment (79). Zocco et al. compared five DCE-US functional parameters (peak intensity, PI; time to PI, T_P_; area under the curve, AUC; slope of wash in, P_w_; mean transit time, MTT) between sorafenib responders (CR+PR+SD) and non-responders (PD) and found a strong correlation between three parameters (AUC, PI and P_w_) on day 15 after treatment with tumor response (80).

#### 4.1.2 Contrast-enhanced computed tomography

Because of the abundance of vascularity within HCC, the tumor could be obviously enhanced on CE-CT imaging. Colagrande et al. investigated the predictive role of the volume of enhancement of disease (VED, defined as volume lesion × arterial enhancement coefficient/volume lesion) on the efficacy of sorafenib and found that clinical benefit patients had a significantly higher rate of VED_T0_ (VED before treatment) > 70% than PD patients (81). Similarly, Nakamura et al. found that patients with pretreatment arterial perfusion (Pre-AP) of HCC on CE-CT higher than 71.7 mL/min/100 mL owned higher OS rate than those without. It made sense because those tumors that had a VED_T0_ > 70% or a Pre-AP > 71.7 mL/min/100 mL possessed a higher level of vascularization, and consequently were more vulnerable to anti-angiogenic therapy.

#### 4.1.3 Dynamic contrast-enhanced magnetic resonance imaging

DCE-MRI is a noninvasive method to measure tumor blood flow, vascular permeability, and interstitial and intravascular volume changes. The volume transfer constant (K^trans^) on DCE-MRI, reflecting the permeability of vessels, is an endpoint for vascular response evaluation. Hsu et al. discovered that baseline K^trans^ was significantly higher in patients with PR or SD than in patients with PD when treated with sorafenib and metronomic tegafur/uracil (82). Moreover, further analysis showed specifically that patients with vascular response, defined as a 40% or greater decrease in K^trans^ after 14 days of treatment, had significantly higher rates of PR and SD than patients without. Higher baseline K^trans^ reflected richer vascularization within HCC, and greater decrease in K^trans^ after treatment implied better sorafenib effect on tumors. Both were reasonable predictive biomarkers for sorafenib response. In addition, the authors also found a correlation between vascular response and hypertension and HFSR, indicating that hypertension and HFSR may also correspond to the response to TKI treatment, which has been reported in many articles.

### 4.2 Imaging features of immunotherapy response prediction

Immune suppression plays an essential role in HCC progression. Immune suppression mainly results from the exclusion of infiltrating T cells and functional suppression of T cells, the latter usually caused by PD-1/PD-L1 expression on tumor cells or immune cells. Current studies investigating imaging biomarkers to predict immunotherapy response mainly focus on the prediction of T-cell infiltration and the expression of PD-1/PD-L1.

#### 4.2.1 Conventional imaging

##### 4.2.1.1 Contrast-enhanced MRI

In a retrospective study evaluating gadoxetic acid-enhanced MR imaging features on HCC infiltrating CD8 cells and PD-L1 expression, Sun et al. showed that irregular tumor margin (ITM) and peritumoral low signal intensity (PLSI) on hepatobiliary phase images were predictors for PD-L1 positivity, absence of an enhancing capsule (AEC) and PLSI were predictors for CD8^+^ high density, and PLSI and ITM were predictors for both (83). In addition, the combination of PLSI and ITM and the combination of PLSI and AEC were found to be correlated with the response to immunotherapy.

#### 4.2.2 Novel imaging

##### 4.2.2.1 Gd-EOB-DTPA-enhanced MRI

**Figure 2:**
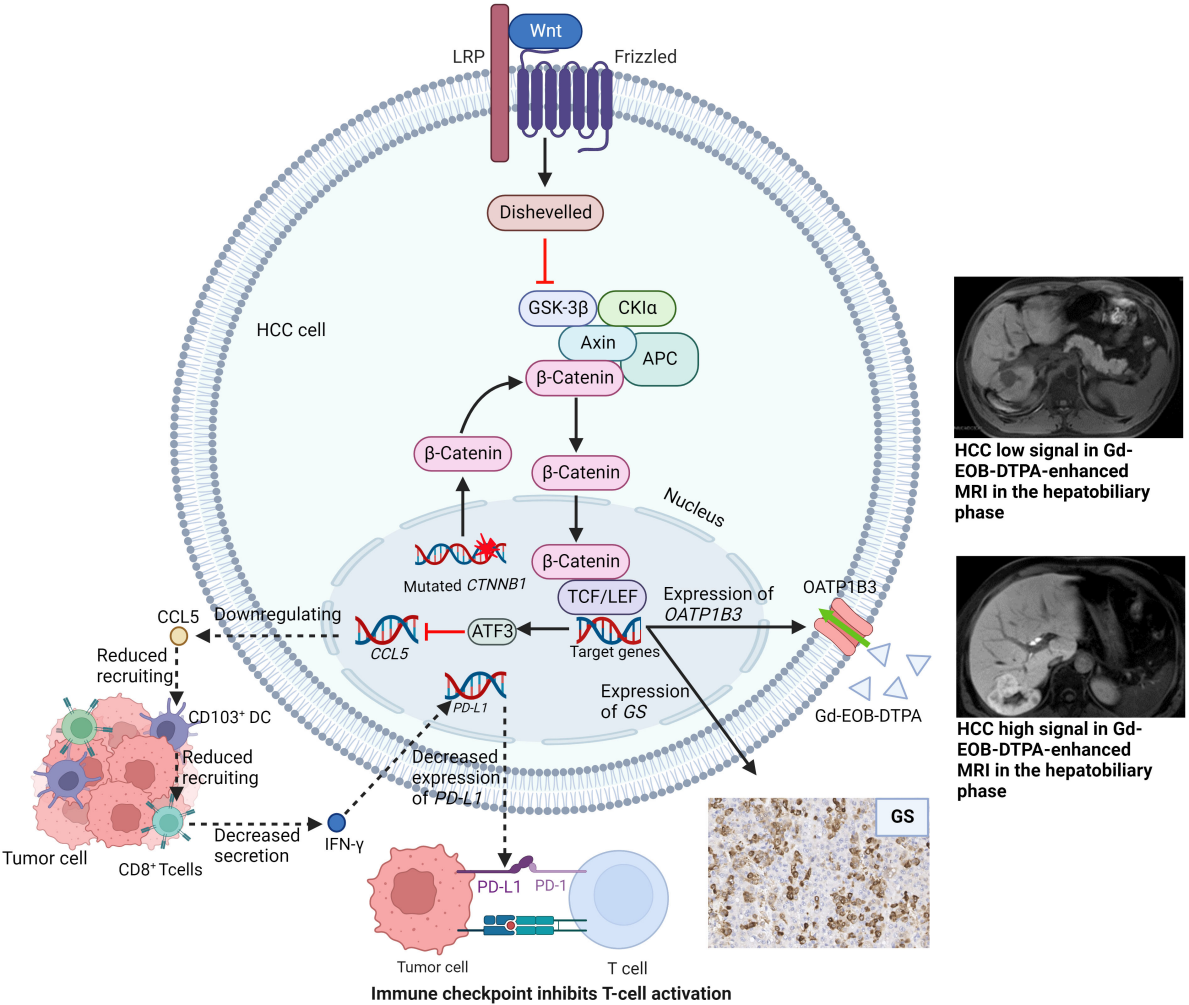
Wnt/β-catenin signaling pathway with related target genes activated and subsequent manifestations. LRP, lipoprotein Receptor-Related Proteins; GSK-3β, glycogen synthase kinase-3beta; CKIα, casein kinase I α; TCF/LEF, T-cell factor/lymphoid enhancer factor; ATF3, activating Transcription Factor; CCL5, C-C chemokine ligand 5; DC, dendritic cells; IFN-γ, interferon-γ; PD-1/PD-L1, Programmed death-1/programmed death-ligand 1; GS, glutamine synthetase; OATP1B3, organic anion transporting polypeptide 1B3.

The activated Wnt/β-catenin signaling pathway is characterized by immune cells exclusion, decreased expression of PD-L1 and increased expression of organic anion transporting polypeptide 1B3 (OATP 1B3). Hepatocytes can take up Gd-EOB-DTPA through OATP 1B3 (84). Given that the Wnt/β-catenin signaling pathway was associated with immunotherapy resistance in HCC, Aoki et al. investigated the predictive role of the hepatobiliary phase of Gd-EOB-DTPA-enhanced MRI on HCC response to immunotherapy (85). The authors found that the ORR, DCR and TTP of hypointense nodules tended to be better than those of hyperintense nodules. It was because that hyperintense lesions were tumors with Wnt/β-catenin signaling activated and resistant to immunotherapy (**Figure 2**). Sasaki et al. had similar results when evaluating HCC patients treated with a combination of atezolizumab plus bevacizumab (86).

#### 4.2.2.2 Radiomics

Radiomics is a new concept proposed in recent years. It refers to the high-throughput extraction of a large number of imaging features that describe tumor and tumor microenvironment characteristics.

Based on the hypothesis that disparate phenotypes of tumors could be detected by high-dimensional imaging data, Liao et al. developed a radiomics-based score (Rad score) using seven imaging features of CE-CT in patients with HCC (87). The score was found to be associated with the percentage of tumor-infiltrating lymphocytes and PD-1/PD-L1 expression on tumor/immune cells.

Hectors et al. retrospectively analyzed radiomics features of HCC patients by MRI. The results turned out that radiomics features were distinctly related to immune cell markers(CD3, CD68 and PD-L1). Furthermore, an association between PD-1 mRNA and radiomics was also found (88).

These correlations between radiomics and immune cells/PD-1/PD-L1 indicated its potential role in predicting immunotherapy response of HCC patients. Nevertheless, it needs to be validated in the future. The report by Hectors et al. included patients without treatment before undergoing MRI and Liao et al. excluded patients treated before CT scan. To our knowledge, the radiologic and biological characteristics of HCC may be alterable during treatment. Patients who are deemed to be appropriate candidates for immunotherapy before treatment might not benefit from the cure after receiving a period of remedy. In this setting, the delta radiomics, which is capable of capture the quantitative changes of tumor radiomic features on treatment going (89), may help clinicians better screen out the proportion of patients suitable for treatment continuation. Similar clinical research has been conducted on rectal cancer and high-grade soft-tissue sarcoma (90, 91). However, to our knowledge, report about the predictive value of delta radiomics on HCC treated with TKIs or immunotherapy has not been published.

Imaging biomarkers serve as a noninvasive method to help select an appropriate HCC patient proportion sensitive to systemic therapies, which is convenient to perform and poses nearly no threat to patient safety. However, the remaining problems are that the results might be influenced by the different precisions of imaging devices and different radiologists in clinical practice. These problems may be solved with the development of artificial intelligence.

## 5 Tumor tissue biomarkers

Biomarkers from tumor tissue at different levels, including DNA, RNA, proteins and cells, from our perspective, are the most accurate predictors of treatment response. These biomarkers are able to directly reflect tumor characteristics and can help to screen liver cancer patients suitable for systemic therapy more accurately.

### 5.1 Proteins within tumor tissue

The mitogen-activated protein kinase (MAPK) signaling pathway, including extracellular signal-regulated kinase (ERK), c-Jun N-terminal kinase (JNK), p38MAPK and ERK5, regulates the inflammatory response and participates in tumorigenesis and angiogenesis of HCC (92, 93).

In human HCC, the abnormal activation of ERK signaling could be frequently observed. The Raf/MAPK ERK kinase (MEK)/ERK pathway is one of the targets of sorafenib (**Figure 1**). Based on a phase II study of sorafenib in advanced HCC patients, Abou-Alfa et al. found that patients with higher pretreatment expression of phosphorylated ERK (pERK) within tumor cells had significantly higher TTP than patients with lower expression of pERK (94). Chen et al. had a consistent result with Abou-Alfa et al. that higher pERK and VEFGR expression was associated with increased TTP (95).

Yamauchi et al. observed that positive immunohistochemistry for fibroblast growth factor receptor (FGFR4) in biopsy samples before treatment was associated with a longer progression-free survival (2.5 *vs.* 5.5 months, P 5 0.01) and a favorable objective response rate treated with lenvatinib, but this association was not observed with blood soluble FGFR4 (96).

For PD-1/PD-L1 inhibitors, the predictive role of PD-L1 expression level was the most commonly investigated. Logically, patients with higher expression levels of PD-1/PD-L1 respond better to PD-L1 inhibitors, which has been reported in many types of cancers, such as lung cancers (97) and melanoma (98). The predictive role of PD-L1 has also been investigated in HCC.

In the KEYNOTE-224 clinical trial, Zhu et al. found that the objective response to pembrolizumab was associated with PD-L1 expression (11). In a randomized phase 2 trial, Qin et al. also showed that the ORR of camrelizumab was significantly higher in HCC patients with expression of PD-L1 ≥1% than in patients with PD-L1 <1% (99). However, patients with negative PD-L1 expression have also been reported to respond to ICIs (10, 100). Moreover, another remaining problem is whether the expression of PD-L1 on tumor cells or immune cells or on both cells represents the selection of sensitive patients to ICIs needs further investigation because the associations between response and PD-L1 expression on both cells have been reported (11, 99, 101–103).

In addition, combined immunotherapy has been proposed in recent years. The results from IMbrave150 study showed that atezolizumab plus bevacizumab therapy was superior to sorafenib monotherapy (104). And a phase IB study reported that combination of pembrolizumab and lenvatinib provided better efficacy than immunotherapy alone (105). These phenomena might be due to that VEGF and FGF suppressed interferon gamma secretion and T cell cytotoxicity, while upregulating the expression of PD-1 (106). Therefore, by targeting VEGF and FGF, the efficacy of immunotherapy was synergeticly enhanced. Additionally, Zhu et al. suggested that anti-VEGF agents might strengthen the antitumor activity of immunotherapy through targeting myeloid cell inflammation and inhibiting the angiogenesis within tumors (107). Hence, predictive biomarkers for combined immunotherapy are worthy of being investigated. Zhu et al. found that HCC with high expression of PD-L1, VEGFR and infiltrating CD8+ T cell had better benefit from atezolizumab plus bevacizumab therapy than monotherapy (107), indicating that HCC patients with these features probably appropriate to combined immunotherapy.

### 5.2 Gene alterations

#### 5.2.1 Molecular targeted therapy

Alterations in genetic levels are essential for the formation of tumor phenotypes and features observed by examinations or in the clinic. The difference in genetic alterations has greatly contributed to the heterogeneity of HCC. Assessment of response prediction for TKIs using genes has been performed by many researchers.

Arao et al. showed that the amplification of *FGF3/FGF4* was observed in three of ten HCC samples from patients who responded to sorafenib, while no amplification of *FGF3/FGF4* was found in 38 patients with SD or PD (21).

With the help of next-generation sequencing, Harding et al. found that HCC patients with activating mutations in the PI3K–mTOR pathway in tumors had significantly lower DCR and shorter PFS and OS after sorafenib treatment (108).

#### 5.2.2 Immunotherapy

HCC can be divided into different molecular subtypes, each with its own genetic mutational signature and distinct phenotype. Calderaro et al. summarized the phenotypic and molecular features of HCC (109). With different mutated genes, HCC subtypes hold different activated signaling pathways and different immune cell infiltration. Thus, HCC subtypes may be capable of helping screen patients suitable for immunotherapy. Of all the subtypes, notably, CTNNB1-mutated HCC with activating WNT/β-catenin pathway has a well-differentiated tumor phenotype and lacks immune cell infiltration (**Figure 2**).

In a prospective study aimed at matching HCC patients to molecular targeted therapy and immunotherapy, Harding et al. proposed that patients with WNT/β-catenin pathway activation treated with ICIs had a lower DCR than patients without (108). This result might be explained by the lack of immune cell infiltration in *CTNNB1*-mutated HCC. Oversoe et al. showed that the integrated analysis of circulating tumor DNAs and DNAs in tumor tissue could improve the detection rate of *CTNNB1* mutation in HCC patients (110), indicating that the combined analysis of tumor tissue and blood might better select HCC patients suitable for immunotherapy.

### 5.3 Infiltrating immune cells in the tumor microenvironment

The antitumor effect of ICIs relies on immune cell infiltration. In the tumor microenvironment, the infiltration of immune cells has been shown to be predictive of the response to ICIs, especially CD8+ cytotoxic T cells. The CheckMate 040 trial found that in HCC patients treated with nivolumab, CD3+ T cells were associated with response (10). Kaseb et al. found that in HCC patients receiving combination therapy with nivolumab and ipilimumab, CD8+ cell infiltration was significantly positively associated with clinical response (111). Ng et al. found that patients with higher levels of CD38+ macrophage infiltration had a higher overall response rate when receiving immunotherapy than those with lower levels of CD38+ macrophages, which may be related to the secretion of IFN-γ by macrophages (112). **Table 3** summarized predictive biomarkers within tumors.

**Table 3:** Predictive biomarkers within tumors for the systemic treatment of hepatocellular carcinoma.

Tumor biomarkers	Reference	Hazard ratio (95% confidence interval)			Positive or Negative
		PFS, TTP or PD	OS	Cut-off	
Proteins					
pERK	Chen et al. (95)	1.504 (1.292-1.217)	NA	NA	Positive for sorafenib
VEGFR	Chen et al. (95)	0.284 (0.411-1.109)	NA	NA	Positive for sorafenib
FGFR4	Yamauchi et al. (96)	0.30 (0.13–0.69)	NA	NA	Positive for lenvatinib
PD-L1	Qin et al. (99)	NA	NA	NA	Positive for camrelizumab
Genes					
*FGF3/FGF4 amplification*	Arao et al. (21)	NA	NA	NA	Positive for sorafenib
*CTNNB1*	Harding et al. (108)	NA	NA	NA	Negative for immunotherapy
Infiltrating immune cells	Ng et al. (112)	0.384 (0.193 to 0.765)	0.463 (0.232 to 0.926)	5% positivity of total immune infiltrates being CD38+ cell	Positive for immunotherapy

PFS, Progression-free survival; TTP, Time to progression; PD, Progressive disease; OS, Overall survival; pERK, phosphorylated extracellular signal-regulated kinase; VEGFR, vascular endothelial growth factor receptor; FGFR, fibroblast growth factor receptor; PD-L1, programmed death-ligand 1. NA, Not available.

## 6 Conclusion

The advent of systematic therapy offers a new management strategy for HCC. However, the reality is that only a small fraction of patients respond and improve survival with targeted therapy and immunotherapy. Predictive biomarkers for treatment response have been investigated, but until now, no biomarkers have been approved to guide treatment decisions. Clinical traits of patients, such as adverse events, are easily available. These features may represent an extrinsic manifestation of the drug’s action and could aid in the selection of patients who respond to treatment at an early stage. Circulating biomarkers, including tumor markers, inflammatory markers, circulating DNAs and tumor cells, serve as noninvasive methods for response prediction. Biomarkers within tumor tissues represent the direct detection of the intrinsic characteristics of tumors. In our opinion, these biomarkers are the most valuable. However, due to the unrequired biopsy for the diagnosis of HCC, it is difficult for physicians and surgeons to obtain tumor tissue before treatment. Here, the necessity of HCC biopsy before treatment is advocated to better understand the intrinsic features of tumors and help guide therapy selection. Moreover, with the emergence of liquid biopsy, the combination of tumor tissue and circulating tumor cells may help us better understand the intrinsic features of tumors and better recognize different HCC subtypes. A single biomarker is insufficient for response prediction. For example, although studies have shown that PD-L1-positive patients respond better to PD-1/PD-L1 blockade, some PD-L1-negative patients respond to the treatment. Future research should also focus on predictive models or scores consisting of multiple biomarkers that are able to combine the predictive values of multiple factors. By this we can maximize the screening of suitable patients for systemic treatment. In clinical practice, patients receive a combination of targeted therapy and immunotherapy; thus, seeking biomarkers predictive of response to the combination therapy would be of more practical significance.

## Author contributions

HW and KX contributed to the conception of the study; PH and HW search the database. PH made the figures and tables; PH, HW and JW wrote this paper; PH, HW and KX revised this paper. All authors contributed to the article and approved the submitted version.

## Conflict of interest

The authors declare that the research was conducted in the absence of any commercial or financial relationships that could be construed as a potential conflict of interest.

## Publisher’s note

All claims expressed in this article are solely those of the authors and do not necessarily represent those of their affiliated organizations, or those of the publisher, the editors and the reviewers. Any product that may be evaluated in this article, or claim that may be made by its manufacturer, is not guaranteed or endorsed by the publisher.
